# Detection of Bovine Leukemia Virus in Bone Marrow of Patients with B-Cell Precursor Acute Lymphoblastic Leukemia: A Case–Control Study

**DOI:** 10.3390/v18030342

**Published:** 2026-03-11

**Authors:** Kerlimber Núñez-Gutiérrez, José Fuentes-Montoya, Leonardo Enciso, Jairo Jaime, Adriana Corredor-Figueroa

**Affiliations:** 1 Universidad Nacional de Colombia, Sede Bogotá, Facultad de Medicina Veterinaria y de Zootecnia, Departamento de Salud Animal, Centro de Investigación en Infectología e Inmunología Veterinaria—CI3V, Cra. 30 # 45-03, Bogotá 111321, Colombia; kenunezg@unal.edu.co; 2 Laboratorio de Investigación en Ciencias Biológicas (LICiB), Universidad ECCI, Bogotá 111311, Colombia; 3 Grupo de Investigación INDEVOS, Facultad de Ingeniería, Universidad EAN, Bogotá 111321, Colombia; jamontoya.d@universidadean.edu.co; 4 Hospital Universitario Nacional, Bogotá 111321, Colombia; ljencisoo@unal.edu.co; 5 Facultad de Medicina, Universidad Nacional de Colombia, Sede Bogotá, Bogotá 111321, Colombia

**Keywords:** bovine leukemia virus, acute lymphoblastic leukemia, B cells, zoonosis, retrovirus, bone marrow

## Abstract

Bovine leukemia virus (BLV) is an oncogenic deltaretrovirus that infects B cells, and its possible presence in humans has garnered increasing attention. This study included 58 participants: 11 with B-cell precursor acute lymphoblastic leukemia (B-ALL, cases) and 47 healthy individuals (controls). Researchers assessed anti-gp51 antibodies and BLV proviral DNA in bone marrow and blood samples. Seropositivity was observed only in the B-ALL group (18.2%; 2/11), while all controls were seronegative. Quantitative PCR targeting the *pol* gene detected proviral DNA in 74.1% of samples, with similar detection rates between cases and controls. Although proviral load was higher in controls, this difference did not reach statistical significance. Conventional and nested PCR for other viral genes revealed a differential pattern: amplification of the *tax* gene was significantly associated with B-ALL, whereas *gag* and *env* were not. Bayesian Chow–Liu network analyses identified dependencies among viral genes and suggested that contextual factors, such as fieldwork, may influence the association between molecular positivity and B-ALL. Sequence analyses showed that the detected BLV strains clustered with previously reported bovine and human sequences from Colombia, all within genotype 1. These findings support human exposure to BLV and raise important questions about its persistence and potential connections to hematological diseases in humans.

## 1. Introduction

Bovine leukemia virus (BLV) is an oncogenic exogenous retrovirus that serves as the causative agent of enzootic bovine leukosis (EBL). After introduction into a herd, BLV infection typically follows a prolonged asymptomatic phase, lasting between 4 and 10 years [1]. Approximately 30% of infected cattle develop a persistent lymphocytosis, while a smaller proportion—between 5% and 10%—progress to more severe disease, such as lymphosarcoma or lymphocytic leukemia [1,2]. BLV remains widely distributed worldwide, with high prevalence rates in endemic regions and phylogenetic diversity across circulating genotypes [3].

BLV is a member of the family *Retroviridae*, genus *Deltaretrovirus*, and shares both genetic and functional characteristics with the human T-cell lymphotropic virus (HTLV) [4,5,6,7]. The BLV genome comprises a positive-sense single-stranded RNA (ssRNA) molecule that is reverse-transcribed by the viral reverse transcriptase into double-stranded DNA (dsDNA), which is subsequently integrated into the host genome [8]. The BLV ssRNA genome is 8714 nucleotides (nt) long and contains eight open reading frames (ORFs), flanked by long terminal repeats (LTRs), which are crucial for both reverse transcription and viral genome integration [8,9]. As with all retroviruses, BLV contains three major genes: *gag*, *pol*, and *env*. The *gag* gene encodes the structural capsid proteins p15 and p24, while the *pol* gene encodes the enzymes reverse transcriptase (RT), integrase (IN), and protease (PR). The *env* gene encodes the envelope glycoproteins gp30 and gp51 [10], which are essential for viral entry by mediating interaction with the cellular receptor CAT1/SLC7A1 [11]. While the *env* gene encodes proteins with structurally conserved regions critical for viral function, it also contains variable domains, making it the primary target for molecular detection and BLV genotyping [12]. As a result, *env* sequencing is extensively employed for BLV phylogenetic classification and has facilitated the identification of at least 12 genotypes [9,13]. A unique feature of the BLV genome is the presence of the pX region, situated between *env* and the 3′ LTR [14]. This region encodes several regulatory and accessory proteins, including Tax, Rex, R3, and G4. Collectively, these proteins play key roles in viral replication, immune evasion, cellular proliferation, gene regulation, and cellular transformation through modulation of multiple signaling pathways [5,9]. A prominent example is the Tax protein, which functions as an initiator of cellular transformation in both BLV and HTLV. Unlike a classical captured oncogene, Tax acts as a viral factor that induces hyperproliferation, deregulates cell-cycle checkpoints, and promotes genomic instability. These effects are mediated, in part, by persistent activation of signaling pathways such as NF-κB and by interference with DNA repair mechanisms, resulting in a mutator phenotype conducive to the accumulation of early genetic alterations [15,16,17,18,19,20,21,22,23]. Consequently, the *tax* gene is routinely utilized to confirm BLV infection, as both its presence and expression have been documented in infected animals and are linked to latent and productive infections [24]. Additionally, the *tax* gene sequence is frequently employed in phylogenetic analyses [25,26]. In contrast to the relatively conserved *pol* gene, *tax* displays greater sequence variability, making it especially valuable for characterizing distinct viral strains [27].

BLV primarily infects CD5^+^ B cells that express surface immunoglobulin M (IgM), but it can also persist in monocyte/macrophage lineage cells [28,29,30]. Viral entry occurs through the interaction of BLV envelope glycoproteins with the cellular receptor CAT1/SLC7A1, a protein highly conserved among mammals [11]. Additionally, vesicular transport proteins such as AP3D1 are thought to participate in later stages of the BLV replicative cycle [31]. Although natural BLV infection is most commonly observed in cattle, it has also been reported in other ruminant species, including buffaloes, sheep, and goats [32,33,34]. Furthermore, in vitro studies have demonstrated that BLV is capable of infecting cells from multiple species, including humans. The virus also infects various cell types, such as mammary epithelial and immune cells [35,36,37,38,39,40,41]. This broader host range and expanded cellular tropism raise concerns about its potential to infect humans. The precise route of transmission to humans remains unclear. Proposed mechanisms include direct contact with infected animals [42], exposure to contaminated biological fluids [43], and ingestion of bovine-derived products like milk and meat, in which viral DNA has been detected [44,45]. Several studies have identified BLV proviral DNA, viral proteins, and anti-BLV antibodies (Abs) in human samples, including those from women with and without breast cancer [46,47,48,49,50,51,52,53,54,55,56,57], lung tissue [58], and blood cells [43,59]. These findings have prompted the hypothesis that BLV could be a risk factor or cofactor in the development of human cancers, particularly epithelial neoplasms. While no conclusive evidence currently establishes a causal relationship between BLV and human disease, these observations indicate that the virus may cross the species barrier and establish persistent infections in humans.

B-cell precursor acute lymphoblastic leukemia (B-ALL) in humans is characterized by the clonal proliferation of B lymphoblasts in the bone marrow [60]. Its etiology is multifactorial, involving both genetic and environmental factors. Certain viral infections have been proposed as cofactors in the malignant transformation of precursor cells [61,62,63,64]. Substantial evidence indicates that various viruses play important roles in cancer development. They act as exogenous agents or as initiators of cellular transformation [65]. An estimated 15–20% of cancer cases in humans and animals are associated with viral infections. These are considered potential risk factors, and in some cases, they have been shown to have causal relationships [66]. Viruses such as hepatitis B virus (HBV), hepatitis C virus (HCV), Epstein–Barr virus (EBV), human papillomavirus (HPV), human T-cell lymphotropic virus type 1 (HTLV-1), and human herpesvirus 8 (HHV-8) have been causally associated with specific cancer types in humans [67]. Certain features of BLV, including its tropism for B cells, its demonstrated ability to infect human cells in vitro, and its zoonotical potential, support the hypothesis of its possible presence in human bone marrow. However, no studies to date have reported the detection of BLV in human bone marrow samples or its potential association with hematological neoplasms such as B-ALL. Therefore, the present study aimed to detect BLV in bone marrow samples from patients with B-ALL and healthy individuals using molecular and serological methods. This research seeks to contribute to the understanding of BLV’s potential role in the pathogenesis of human diseases.

## 2. Materials and Methods

### 2.1. Study Design

This analytical, observational case–control study employed a post hoc approach. A total of 58 individuals were included: 11 with a confirmed diagnosis of B-ALL and 47 controls without B-ALL. Participants were recruited between June 2022 and June 2024 from two tertiary care hospitals in Bogotá, Colombia: Hospital Universitario Clínica San Rafael and Hospital Universitario Nacional de Colombia. All patients considered for bone marrow aspiration during the study period were eligible. Based on flow cytometry findings, participants were classified as cases (B-ALL-positive, *n* = 11) or controls (negative for any hematological disease, *n* = 47). Those diagnosed with other hematological neoplasms or conditions unrelated to B-ALL were excluded.

Prior to sample collection, a structured questionnaire was administered to identify epidemiological risk factors associated with BLV exposure. Subsequently, trained clinical staff at each participating hospital collected bone marrow aspirates and peripheral blood samples in accordance with established clinical protocols. All samples were stored at 4 °C and promptly transported to the Biosafety Level 3 (BSL-3), Laboratorio de Investigación en Ciencias Biológicas at Universidad ECCI in Bogotá, Colombia. Upon arrival at the laboratory, serum was separated from peripheral blood samples and stored at −20 °C until ELISA analysis. Bone marrow aspirates were subjected to density gradient centrifugation to isolate mononuclear cells, which were subsequently stored at −80 °C for later nucleic acid extraction and PCR-based analyses.

### 2.2. Serological and Molecular Detection of BLV

#### 2.2.1. Sample Processing, Serological Analysis, and DNA Extraction

Serum was separated from peripheral blood samples by centrifugation at 1500× *g* for 10 min using a Medifuge™ centrifuge (Thermo Fisher Scientific, Waltham, MA, USA). Detection of anti-gp51 BLV Abs was performed using a blocking ELISA kit (NGEZIM BLV COMPAC 2.0, ref. 12.BLV.K3, Ingenasa, Madrid, Spain) according to the manufacturer’s instructions.

Bone marrow samples were processed by density gradient centrifugation using LymphoSep™ (Biowest, Nuaillé, France) to isolate mononuclear cells. Genomic DNA was subsequently extracted with the High Pure PCR Template Preparation Kit (Roche Diagnostics GmbH, Mannheim, Germany). DNA concentration and purity were assessed using a NanoDrop OneC spectrophotometer (Thermo Fisher Scientific, Waltham, MA, USA). DNA integrity and the absence of PCR inhibitors were verified by amplification of the housekeeping gene GAPDH (glyceraldehyde-3-phosphate dehydrogenase), employing primers previously reported by Buehring et al. (2014) [49], which have been validated for BLV-related studies in human tissues. Only samples positive for GAPDH amplification were used for further molecular analyses.

#### 2.2.2. Molecular Detection of BLV

Detection of BLV proviral DNA was carried out using quantitative PCR (qPCR) targeting the pol gene. The assay utilized PerfeCTa qPCR ToughMix (Quantabio, Gaithersburg, MD, USA), an FAM–BHQ-labeled TaqMan probe, and specific primers (Table 1). Primer and probe sequences were verified for specificity by BLASTn (NCBI BLAST, National Center for Biotechnology Information, Bethesda, MD, USA) analyses (http://blast.ncbi.nlm.nih.gov/Blast.cgi, accessed on 23 March 2023) against the nt collection database. This database included sequences from exogenous viruses and human endogenous retroviruses. Parameters were optimized to ensure high sequence similarity. For assay standardization and proviral load quantification, serial dilutions of a recombinant plasmid containing a fragment of the BLV pol gene were employed. This plasmid was generously provided by the Virology Laboratory, Faculty of Veterinary Sciences, UNCPBA-CONICET (CIVETAN)–CICPBA, Tandil, Argentina. qPCR reactions were performed on a CFX96 real-time PCR system (Bio-Rad Laboratories, Hercules, CA, USA) under previously standardized conditions (Table 1). Each run included both positive and negative controls. Proviral loads were calculated from the standard curve and reported as copies/µL of extracted DNA. Results were interpreted based on amplification curves and threshold cycle (Ct) values.

For detection of the *gag* gene, a conventional PCR was performed to amplify a 385 bp fragment using primers previously described by Buehring et al. (2014) [49]. In a similar manner, the *env* gene was detected by nested PCR with primers reported by Mendoza et al. (2024) [43], which amplify a 444 bp fragment. Specifically, the first PCR round was carried out in a final volume of 10 μL containing 50 ng of DNA, 0.625 μL of each primer (10 μmol/L), and 5 μL of Taq 2X PCR Mix (ABclonal, Woburn, MA, USA). Subsequently, for the second round, 3 μL of a 1:10 dilution of the first-round PCR product was used with the same reagent concentrations and a final volume of 10 μL. Thermal cycling conditions consisted of an initial denaturation at 94 °C for 5 min, followed by 35 cycles of 94 °C for 30 s, annealing at 63 °C (first round) or 62.6 °C (second round) for 30 s, and extension at 72 °C for 1 min, with a final extension at 72 °C for 5 min. Furthermore, amplification of the *tax* gene was also performed by nested PCR using primers previously described by Buehring et al. (2014), which amplify a 206 bp fragment [49]. The reaction conditions and volumes were similar to those described for the *env* gene, with primer-specific concentrations indicated in Table 1. Lastly, PCR products were visualized by electrophoresis on 1% agarose gels using a 100 bp HyperLadder™ molecular weight marker (Meridian Life Science Inc., USA) and EZvision™ staining (AMRESCO Inc., Solon, OH, USA) and documented using a Gel Doc XR+ system (Bio-Rad Laboratories, Hercules, CA, USA).

Serological and molecular markers were analyzed independently to ensure consistent interpretation. Seropositivity by ELISA indicated prior BLV exposure through the presence of anti-gp51 Abs. Detection of the *pol* gene by qPCR identified proviral DNA, whereas amplification of *gag*, *env*, and/or *tax* genes by conventional or nested PCR confirmed specific viral sequences. A sample was considered “BLV positive” if any molecular marker (*pol*, *gag*, *env*, or *tax*) was detected, regardless of serological status. Combined marker profiles were evaluated to assess concordance and to explore potential biological scenarios, such as active infection, defective provirus, or previous exposure without detectable molecular evidence.

### 2.3. Data Analysis

#### 2.3.1. Sequence Analysis and Molecular Phylogeny

Samples showing the expected amplicon sizes for the BLV *env*, *gag*, and *tax* genes were selected for bidirectional Sanger sequencing at SSIGmol (Servicio de Secuenciación y Análisis Molecular, Instituto de Genética, Universidad Nacional de Colombia, Bogotá). Resulting sequences were edited and assembled using Geneious^®^ software version 9.1.2 (Auckland, New Zealand). Sequences generated in this study were then aligned with reference sequences from GenBank, representing the 12 BLV genotypes reported worldwide. For the *gag* gene, alignments included sequences from bovine isolates from Colombia and other countries, as well as human-derived sequences reported in previous studies. The env gene sequence obtained was compared against reference sequences for all 12 genotypes. Finally, *tax* gene sequences were aligned with bovine genotype sequences and with sequences derived from sheep, humans, and consumer products (milk and meat) previously reported by our group [34,44,52,69]. Multiple sequence alignments were performed using the Clustal W algorithm in MEGA version 11 [70] with default automatic settings. Alignments were manually inspected to ensure accurate nt placement. Phylogenetic analyses were conducted under a maximum likelihood (ML) framework using IQ-TREE version 3.0.1 [71]. The optimal evolutionary model for each dataset was automatically selected with ModelFinder [72]. Branch support was assessed using the ultrafast bootstrap (UFBoot) method with 1000 replicates [73]. Finally, phylogenetic trees were visualized and edited with the Interactive Tree of Life (iTOL) platform, version 6.

#### 2.3.2. Statistical Analysis

Continuous variables were summarized as mean ± standard deviation (SD) or as median and interquartile range (IQR), while categorical variables were presented as absolute and relative frequencies (*n*, %). Associations between B-ALL status (case/control) and binary markers (serology, *pol* gene, *gag* gene, *tax* gene, and *env* gene) were evaluated using beta-binomial models with Jeffreys prior [74], yielding posterior distributions for positivity probabilities and odds ratios (ORs). Proviral load, expressed as log_10_ copies/µL, was analyzed using a Bayesian model with Student’s t likelihood and weakly informative priors for group-level parameters [75,76] Between-group differences were reported as Δ = μ_cases − μ_controls, along with the corresponding 95% credible interval (95% CrI) [77]. Additionally, Chow–Liu dependency networks [78] were estimated for discrete variables. Virological variables included detection of the *pol*, *gag*, *env*, and *tax* genes, ELISA seropositivity, and proviral load, while epidemiological covariates comprised B-ALL status, history of fieldwork, consumption of meat, milk, or other animal-derived products, and direct contact with cattle, sheep, and goats. Networks were inferred using bias-corrected mutual information. Edge stability was assessed by bootstrap resampling (1000 replicates) [79]. Predictive performance was evaluated via 10-fold cross-validation. As complementary analyses, exact ORs were calculated using the Baptista–Pike method [80]. Firth’s penalized logistic regression was applied in cases of complete or quasi-complete separation [81,82], and *p*-values and Benjamini–Hochberg false discovery rate (FDR) adjustments [83]. All analyses were performed in Python version 3.11 [84]. The libraries pandas [85], NumPy [86], SciPy [87], matplotlib and seaborn [88,89], and networkx [90] were utilized. Reproducibility was ensured by a fixed random seed.

## 3. Results

### 3.1. Sociodemographic Characteristics and Exposure Variables of the Study Population

A total of 58 participants were enrolled, comprising 11 B-ALL cases and 47 controls, all recruited from two healthcare institutions in Bogotá. The sex distribution was nearly equal, with 51.7% female and 48.3% male participants. The control group’s mean age was 59.2 ± 19.8 years (range: 19–90), while B-ALL patients had a mean age of 35.4 ± 14.7 years (range: 20–65). The age breakdown was as follows: 18–28 years, 18 participants (31.0%); 39–49 years, 13 participants (22.4%); and 60–80 years, 19 participants (32.8%). Regarding exposure variables, 36.2% of participants reported direct contact with cattle, sheep, and goats, and among these, 57.1% reported permanent contact. Importantly, no statistically significant associations were found between animal contact and detection of the *pol* gene (OR = 1.79; 95% CI: 0.49–6.58; *p* = 0.535), nor with the consumption of meat, milk, or other animal-derived products.

### 3.2. Serological and Molecular Detection of BLV in Relation to B-ALL

**Figure 1:**
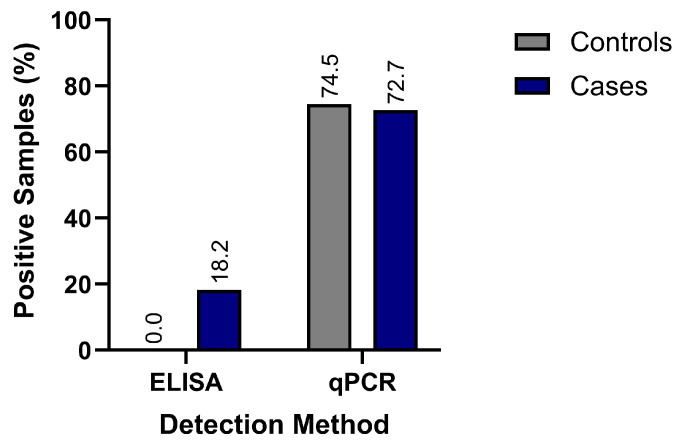
BLV detection by ELISA and qPCR in B-ALL cases and controls. Bars indicate the number of positive samples per group. We found anti-gp51 antibodies by ELISA in 2 out of 11 B-ALL cases but none of the 47 controls. BLV proviral DNA was detected with qPCR (pol gene) in 8 out of 11 B-ALL cases and 35 out of 47 controls.

In the ELISA assay, anti-gp51 Abs were detected in 2 of 11 B-ALL cases (18.2%) and in none of the 47 controls (Figure 1). While Bayesian inference suggested a high posterior probability of OR > 1, the credible intervals were wide, reflecting the limited number of observed events.

BLV proviral DNA was detected by amplifying several molecular markers, including the *pol* gene via qPCR and the *tax*, *gag,* and *env* genes by conventional or nested PCR. qPCR analyses of the *pol* gene identified proviral DNA in 74.1% (43/58) of all samples. Comparison of positivity rates between groups showed no significant differences, with frequencies of 72.7% in cases and 74.5% in controls (Figure 1). Regarding proviral load, controls had higher values (mean: 1.54 × 10^3^ copies/µL; median: 2.59 × 10^2^) than cases (mean: 2.18 × 10^2^ copies/µL; median: 1.18 × 10^2^), though these differences were not statistically significant.

**Figure 2:**
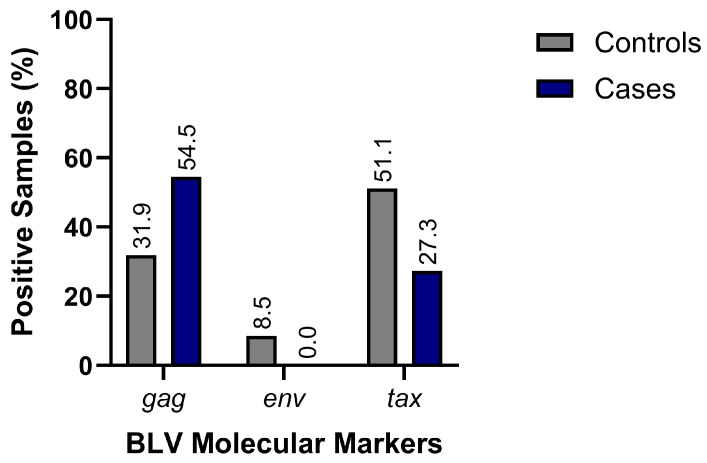
Conventional PCR results showing detection of BLV *tax*, *gag*, and *env* genes in both case and control groups evaluated in this study.

Additionally, the presence of other structural and regulatory BLV genes was assessed by PCR (Figure 2). Bivariate analysis revealed a significant association between *tax* gene positivity and B-ALL status (OR = 2.89; 95% CI: 1.03–8.09; *p* = 0.0487). In contrast, the *gag* gene was detected in 36.2% of total samples, with distributions similar between cases (*n* = 6) and controls (*n* = 15). Meanwhile, the *env* gene was detected exclusively in four control samples and not in any case samples (Figure 2). We found multiple BLV genomic regions in all participants. Specifically, 41% had at least two viral gene fragments, and 59% had three or more. These cases occurred in both study groups, supporting the idea of integrated proviral sequences rather than random amplification events. Finally, per the study’s operational criteria, any sample testing positive for at least one molecular marker was classified as BLV-positive, regardless of serological status.

### 3.3. Association Between B-ALL and BLV Detection Variables

We assessed the association between B-ALL and BLV biomarkers using Bayesian logistic regression models. Among anti-gp51 Abs detected by ELISA, 2 of 11 cases (18.2%) were seropositive, while none of the 47 controls (0%) tested positive. The posterior median odds ratio (OR) of 50.2 [95% credible interval (CrI): 2.38–2.5 × 10^4^] and a posterior probability P(OR > 1) = 0.995 suggest greater seropositivity in cases; however, these estimates were imprecise due to the small number of events. Detection of the *pol* gene showed similar proportions in cases (8/11) and controls (35/47), with a median OR of 0.89 [95% CrI: 0.22–4.29; P(OR > 1) = 0.44], indicating no clear differences between groups. For the *gag* gene, 6/11 cases and 15/47 controls were positive, yielding a median OR of 2.54 [95% CrI: 0.68–9.91; P(OR > 1) = 0.918]. This may suggest higher odds in cases, but the evidence remains uncertain. For nPCR, the *tax* gene was detected in 3/11 cases and 24/47 controls, with a median OR of 0.37 [95% CrI: 0.08–1.42; P(OR > 1) = 0.075], indicating more frequent detection in controls. The *env* gene was observed only in controls (4/47), with a median OR of 0.21 [95% CrI: 4.4 × 10^−4^–3.45; P(OR > 1) = 0.17], but credible intervals were wide. The Bayesian model for proviral load revealed few differences between groups, with the median posterior difference near zero; the 95% CrI included the null value, and the posterior probability P(Δ > 0) was close to 0.5, indicating no pattern of higher proviral load in either group.

### 3.4. Dependency Network (Chow–Liu Model)

**Figure 3:**
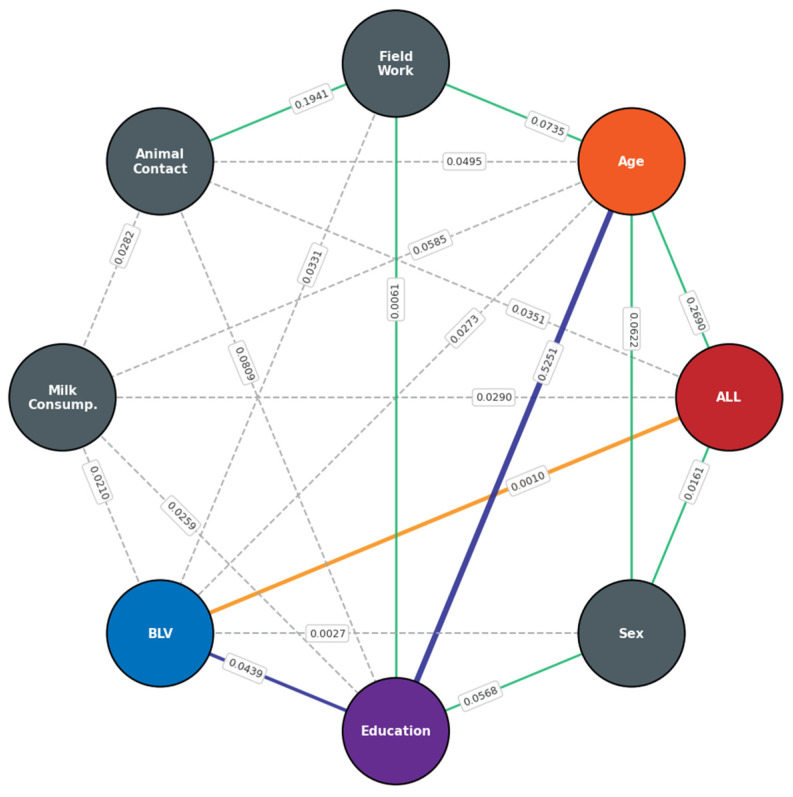
Bayesian dependency network constructed using the Chow–Liu model, illustrating relationships among viral biomarkers and epidemiological covariates. Nodes represent individual variables. Edge thickness and color indicate the strength of dependence: dark blue and green edges indicate the strongest associations; gray dashed edges represent weak or unstable dependencies; the orange edge signifies a statistically significant but low-magnitude association. The connection from “Field work” to other variables highlights its contextual role within the dependency structure. Edges reflect probabilistic associations rather than direct causality.

Analysis using Chow–Liu Bayesian networks allowed exploration of probabilistic relationships among virological variables, including molecular detection of the *pol*, *gag*, *env*, and *tax* genes, ELISA seropositivity, and proviral load, as well as their relationships with the key epidemiological covariates: B-ALL status, history of fieldwork, consumption of meat, milk, or other animal-derived products, and direct contact with cattle, sheep, and goats (Figure 3). The reconstructed network revealed high internal coherence among BLV detection indicators, with strong dependencies between *pol*, *gag*, and *tax* (coefficients > 0.4), suggesting these signals originate from a single infectious agent and reflect a shared underlying process. In the exploratory model, ELISA seropositivity, *tax* detection, and proviral load had the greatest influence on B-ALL status. However, the network showed no direct dependency between B-ALL and BLV detection. In contrast, the variable “fieldwork” occupied a central position in the dependency structure, acting as a potential mediator or confounder that modulates the relationship between BLV exposure and disease status. These findings suggest that occupational and contextual factors may play a key role in viral exposure within the evaluated population.

### 3.5. Viral Sequence Analysis and Identification of BLV Genotypes

**Figure 4:**
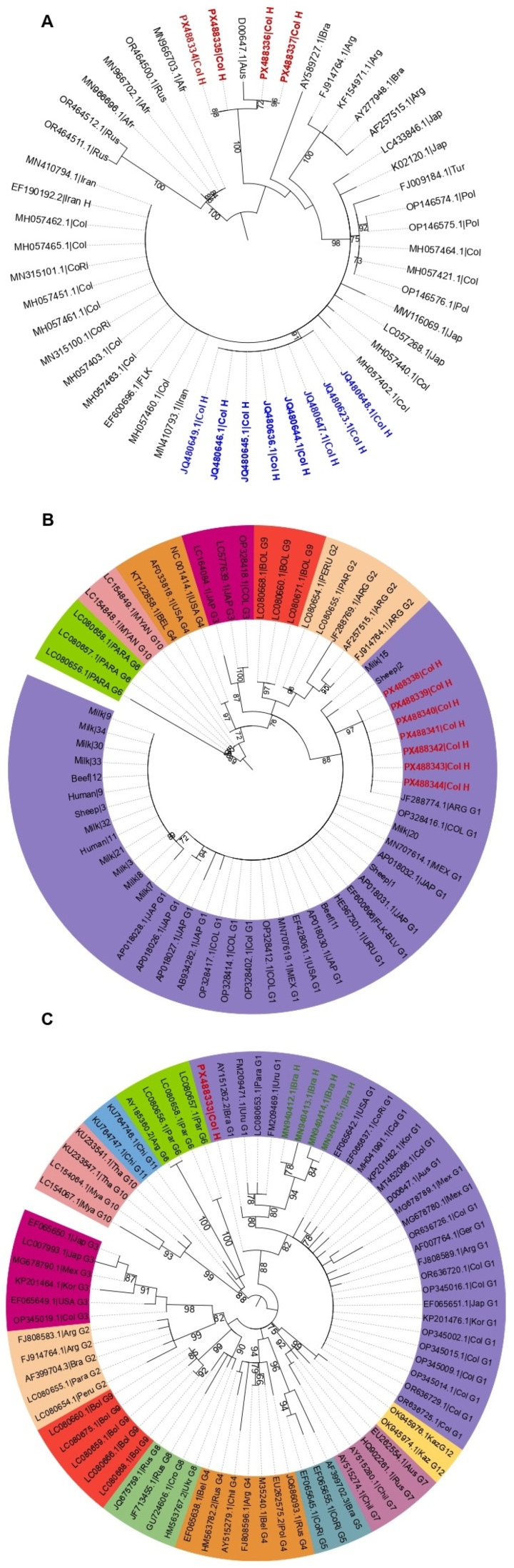
Maximum likelihood (ML) phylogenetic analysis of the BLV *gag*, *env*, and *tax* genes. (**A**) *gag* gene: Phylogenetic tree inferred with HKY+F+I substitution model; branch support values estimated from 1000 bootstrap replicates (values > 70 shown at nodes). Reference sequences are labeled by GenBank accession number and country. Human-derived sequences from this study are shown in red; Colombian studies shown in blue; bovine-derived sequences shown in black. Dataset; 49 sequences, 375 positions. (**B**) *tax* gene: Phylogenetic tree inferred using the TPM2u+I substitution model; branch support from 1000 bootstrap (values >70 shown). Analysis included 62 partial *tax* gene sequences (209 alignment positions). Seven bone marrow *tax* sequences from this study are shown in red. Sequences from consumer products (milk and meat), and Colombian human, bovine, and ovine sources (without GenBank numbers), are shown in black. Genotypes are color-coded. (**C**) *env* gene: Phylogenetic tree inferred using TPM2u+I; branch support from 1000 bootstraps (values > 70 shown). The *env* sequence from this study is shown in red. Previously reported human sequences from Brazil are shown in green. Bovine sequences from different genotypes are shown in black; genotypes are color-coded. Dataset: 76 sequences, 383 positions.

Twelve positive amplicons were sequenced (*env* = 1, *gag* = 4 and *tax* = 7), and all resulting sequences were deposited in GenBank under accession numbers PX488333 to PX488344. MegaBLAST analysis revealed a high nt identity (99.5% to 100%) between these sequences and BLV sequences available in GenBank. Phylogenetic analysis of the *gag* segment showed a close evolutionary relationship between our sequences and BLV strains previously reported in bovines and humans worldwide, with no segregation based on sample origin (Figure 4A). Genotypic assignment using *env* and *tax* genes indicated that sequences from bone marrow samples, both from B-ALL patients and controls, consistently clustered within a monophyletic clade corresponding to BLV genotype 1, alongside reference sequences from bovines and humans (Figure 4B,C). No clustering was observed based on clinical status.

## 4. Discussion

This study is among the first to report BLV detection in human bone marrow samples, especially in individuals with B-ALL. Previous investigations targeted a single genomic region in human hematological samples and reported negative results [91]. In contrast, this case–control analysis detected multiple viral genomic regions (*pol*, *gag*, *tax*, and *env*) using various methodologies. Molecular findings were complemented by phylogenetic and statistical analyses, including Bayesian inference and Chow–Liu network modeling. This approach allowed the exploration of relationships between BLV detection and clinical variables and generated hypotheses about BLV–human host interactions.

The results demonstrate BLV exposure in the studied population, as indicated by the frequent detection of the *pol* gene in both cases and controls. While the positivity rate for *pol* was similar across groups, detection patterns for the *tax*, *gag*, and *env* genes differed: *tax* was predominantly detected in controls, *gag* showed similar frequencies in both groups, and *env* was found exclusively in controls. These findings suggest that, beyond the presence of proviral DNA, there are differences in how specific regions are conserved, detected, or function between individuals with and without B-ALL. This may reflect distinct stages or states of BLV infection.

A growing body of literature supports the presence of human BLV infection. Experimental studies show the virus can infect multiple cell types and cross species [34,37,40,41,59]. Reports from the USA, Australia [47,48,49,50], Egypt [92], Argentina [51], Brazil [54,56], and Colombia [46,52,93] have found Abs, viral proteins, and BLV-DNA (provirus) in samples from healthy people and breast cancer patients. These findings place this study’s results in context and underscore that human exposure to BLV is not uncommon.

In this study, Abs detection by ELISA was limited to individuals with B-ALL. The small number of seropositive samples resulted in substantial statistical uncertainty, reflected by wide credibility intervals in the Bayesian analysis. This pattern aligns with previous reports of low and variable anti-BLV Abs seroprevalence in humans [59,94,95]. By contrast, qPCR detection was much higher, with a 74.1% positivity rate for the *pol* gene in patients from Cundinamarca, similar to the 69% rate reported in Colombian cattle from this same region in 2020 [69]. The discrepancy between these methods may be due not only to differences in analytical sensitivity but also to the presence of defective or transcriptionally silent proviruses. This phenomenon is well-documented in related retroviruses such as HTLV-1, where proviral integration does not always lead to full viral replication [96,97,98]. Studies of adult T-cell leukemia patients have shown proviruses with deletions in structural genes, while regulatory regions such as LTRs and transactivating genes may remain conserved [97]. In this context, the high frequency of pol gene detection without a strong serological response, as seen in our study, suggests BLV proviral DNA may be integrated into the cellular genome without efficient expression of highly immunogenic viral proteins such as gp51. This interpretation should be considered a working hypothesis requiring further functional validation.

The differences in *tax* gene detection between cases and controls offer insights into BLV infection dynamics. The lower *tax* detection in the case group (B-ALL patients) aligns with oncogenic retrovirus models. In these models, regulatory gene expression is favored during early infection to promote cellular proliferation [22]. Later, suppression of these genes may provide a selective advantage to cellular clones that evade immune surveillance [23,96,99,100]. The higher proviral loads in controls may reflect a more severe infection state, with more infected cells and greater proviral transcriptional activity. In contrast, lower proviral loads in cases could indicate clonal expansion of previously transformed cells. In these cells, active viral replication is no longer necessary to maintain the transformed phenotype [99,101]. In cattle, the natural host of BLV, low proviral loads are frequently observed in asymptomatic carriers who remain clinically stable for extended periods, whereas higher burdens are associated with an increased risk of disease progression [102]. Similarly, in this cohort, low proviral DNA copy numbers do not necessarily indicate active replication but may instead reflect a persistent, controlled infection state. In deltaretrovirus infections, viral persistence is maintained not only through de novo infection from viral progeny but also through mitotic expansion of infected lymphocytes [98]. During chronic phases, clonal proliferation may predominate over productive replication, which allows stable maintenance of integrated proviral DNA despite limited viral gene expression [103]. Therefore, the low proviral loads observed in both human cases and controls are consistent with the biology seen in cattle and are biologically plausible. However, extrapolation of proviral load thresholds from the bovine host to humans should be approached with caution because the dynamics of BLV infection in humans remain incompletely characterized.

Bayesian dependency analysis indicated that the relationship between B-ALL and BLV positivity may be influenced by contextual factors, particularly occupational exposure. Fieldwork or contact with production animals emerged as a potential mediator, consistent with previous studies in the same region of Colombia that identified significant associations between molecular BLV detection and occupational exposure to cattle [43]. These results reinforce the hypothesis of possible zoonotic transmission linked to exposure to biological fluids from infected animals.

Phylogenetic analysis of the obtained sequences revealed that all clustered within BLV genotype 1, the genotype most prevalent in cattle in Colombia [69] and previously reported in humans in the country [43]. This genotypic concordance suggests a shared source of exposure and supports the potential for interspecies transmission, likely linked to direct contact with infected animals or the consumption of animal-derived products. Although BLV was detected at a high frequency in the evaluated population, the relatively low proportion of B-ALL cases with molecular evidence of the virus indicates that BLV infection alone is insufficient to induce malignant transformation of B cells. Therefore, our findings should be interpreted within this multifactorial framework, and epidemiological variables such as age also warrant consideration. BLV infection in its natural host is characterized by prolonged latency, with EBL typically developing after several years of persistent infection and affecting only a minority of infected animals. In our study, proviral sequences were identified in both B-ALL patients (mean age: 35.4 ± 14.7 years) and healthy controls (mean age: 59.2 ± 19.8 years). The detection of proviral DNA in older control individuals without hematological malignancy suggests that viral persistence alone is associated with, but does not necessarily cause, leukemogenesis. Conversely, the younger mean age in the B-ALL group does not exclude prior exposure followed by viral persistence; however, it suggests that additional host, viral, or environmental factors may be associated with malignant transformation. Therefore, although age helps assess temporal plausibility, it does not establish causality and must be interpreted within a broader biological and epidemiological context.

Several studies have proposed that retroviruses such as BLV and HTLV-1 can integrate into genomically active regions and alter host gene expression through mechanisms including cis-perturbation [104], induction of genomic instability [15,105,106], and modulation of the immune response [5,16,20,21,22,107,108]. Collectively, the findings of this study further strengthen the evidence supporting the presence of BLV in humans and highlight important questions regarding its biology, persistence, and potential interactions with hematological diseases. However, larger studies utilizing functional approaches are needed to elucidate the specific role of BLV in the pathophysiology of B-ALL and to determine whether its detection represents a marker of exposure, a biological cofactor, or merely an incidental finding within a complex and multifactorial process.

## 5. Conclusions

This study demonstrates the detection of BLV proviral DNA in human bone marrow samples from both individuals with B-ALL and control subjects. The frequency of molecular detection exceeded that of ELISA seropositivity, suggesting scenarios consistent with infections characterized by low antigenic expression or the presence of potentially defective proviruses. Distinct patterns in viral gene detection were observed, particularly a lower frequency of *tax* gene detection and reduced proviral loads among B-ALL cases. These findings suggest alterations in the conservation or expression of specific viral genes during infection; however, it is important to note that the study design does not allow for the establishment of causal relationships between BLV and malignant transformation. The association between BLV presence and B-ALL status may be influenced by occupational factors, especially fieldwork, underscoring the relevance of environmental exposures in risk assessment. Furthermore, the identification of BLV genotype 1, consistent with the predominant genotype found in cattle from the study region, supports a shared source of exposure and the hypothesis of interspecies transmission. In summary, these findings underscore the importance of addressing BLV within an integrated health framework, such as the One Health approach, and justify the necessity for prospective and functional studies to elucidate the biological role of BLV in humans.

## Figures and Tables

**Table 1:** Primer sequences, genomic locations, PCR types, amplicon sizes, and reaction conditions for the detection of genes evaluated in this study.

Gene	Sequence 5′–3′	Location (pb)	PCR Type	Size	Annealing Temperature (°C)	Reference
*GAPDH*	F:GAGTCAACGGATTTGGTCGT	194–213	Conventional PCR	237	50.5	[49]
	R:TTGATTTTGGAGGGATCTCG	431–412				
*pol*	F:CACCATTCACCCCCACTTG	4446–4467	qPCR	183	63	[68]
	R:TCAGAGCCCTTGGGTGTTTC	4628–4605				
	P:CTTCCCATGACTCAGGCCCTTTCT	4536–4559			65	
*gag*	F:AACACTACGACTTGCAATCC	1068–1087	Conventional PCR	385	56	[49]
	R:GGTTCCTTAGGACTCCGTCG	1453–1434				
*env*	F:TCTGTGCCAAGTCTCCCAGATA	5037–5058	nPCR (external round)	598	63	[49]
	R:AACAACAACCTCTGGGGAGGGT	5634–5613				
	F:CCCCAGGCGGCGCCGGTTT	5104–5125	nPCR (internal round)	444	62.6	
	R:GCGAGGCCGGGTCCAGAGCTG	5547–5527				
*tax*	F: CTTCGGGATCCATTACCTGA	7197–7216	nPCR (external round)	373	56.5	[49]
	R:GCTCGAAGGGGGAAGTGAA	7570–7551				
	F:GGCCCCACTCTCTACATGC	7265–7283	nPCR (internal round)	206	60	
	R:AGACATGCAGTCGAGGGAAC	7471–7452				

## Data Availability

The sequence data generated in this study are available in the NCBI GenBank database under accession numbers [PX488333–PX488344]. Other data used and/or analyzed during the present study are available from the corresponding author upon reasonable request.

## Abbreviations

The following abbreviations are used in this manuscript:

BLV	Bovine leukemia virus
B-ALL	B-cell acute lymphoblastic leukemia
EBL	Enzootic bovine leukosis
ssRNA	Single-stranded RNA
dsDNA	Double-stranded DNA
ORFs	Open reading frames
LTRs	Long terminal repeats
HTLV	Human T-lymphotropic virus
Abs	Antibodies
qPCR	Real-time quantitative polymerase chain reaction
nPCR	Nested polymerase chain reaction
OR	Odds ratio
nt	Nucleotides
RT	Reverse transcriptase
IN	Integrase
PR	Protease
IgM	Inmunoglobulin L
HBV	Hepatitis B virus
HCV	Hepatitis C virus
EBV	Epstein–Barr virus
HPV	Human papillomavirus
HHV-8	Human herpesvirus 8
GAPDH	Glyceraldehyde-3-phosphate dehydrogenase
SSIGmol	Servicio de Secuenciación y Análisis Molecular
ML	Maximum likelihood
UFBoot	Ultrafast bootstrap
IQR	Interquartile range
CrI	Credible interval
